# Supplementary figures and images for: Regeneration in the ctenophore Mnemiopsis leidyi occurs in the absence of a blastema, requires cell division, and is temporally separable from wound healing
Source: BMC Biol. 2019 Oct 11;17:80. doi: 10.1186/s12915-019-0695-8 (PMC6788111; doi:10.1186/s12915-019-0695-8)

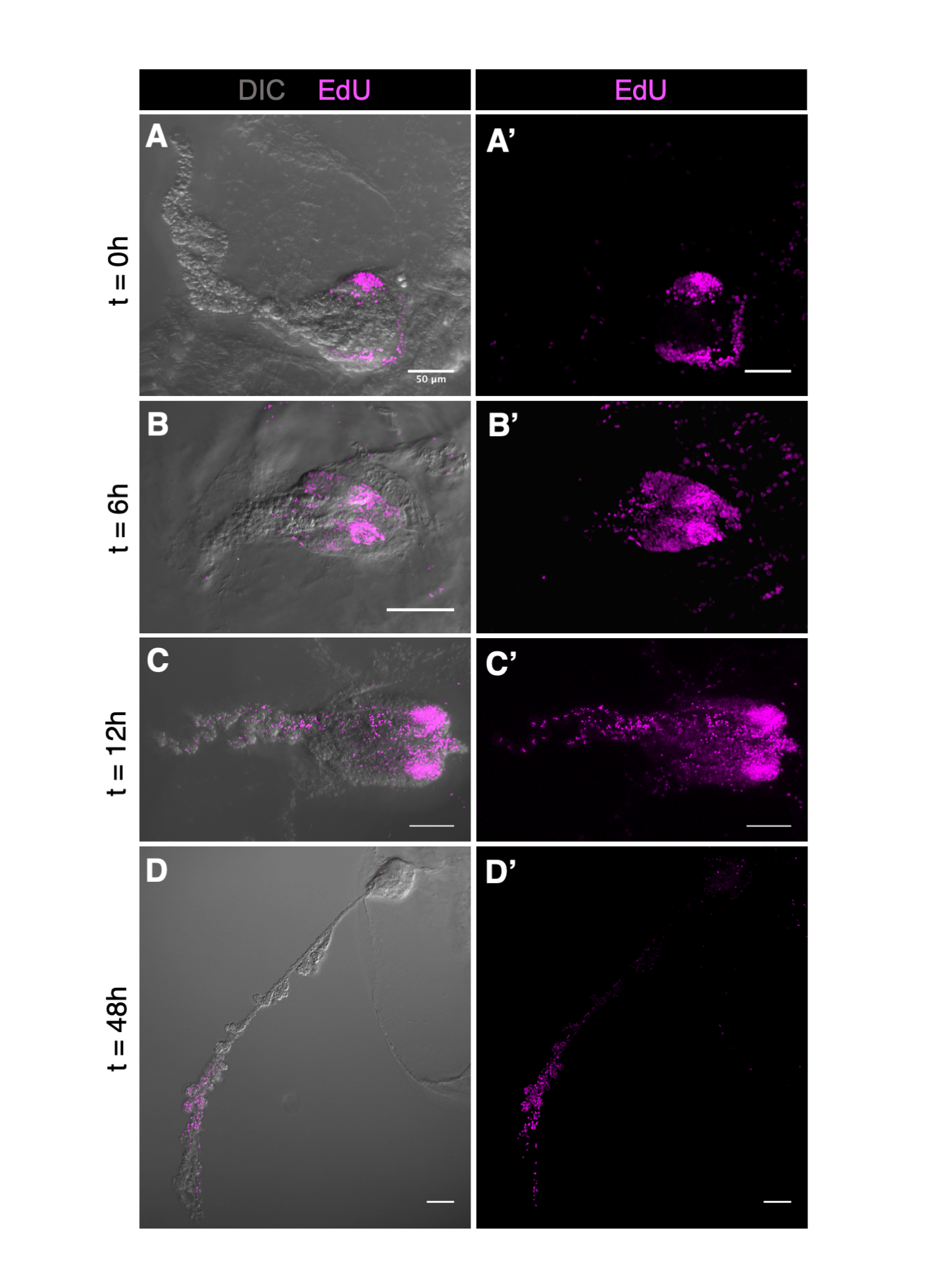

Supplement: Supplementary file 2 — Figure S5. EdU pulse and chase experiment in the tentacle bulb. (A-D’) Confocal stack projections of tentacle bulbs and tentacles in lateral view. The time of the chase is listed to the left of the rows, and the labeling corresponding to each panel is listed at the top of the columns. Nuclei of S-phase cells are labeled with EdU (magenta) combined with a differential interference contrast image of the tissue (DIC). Scale bars = 50 μm. (TIFF 7376 kb) [file 12915_2019_695_MOESM2_ESM.tiff]

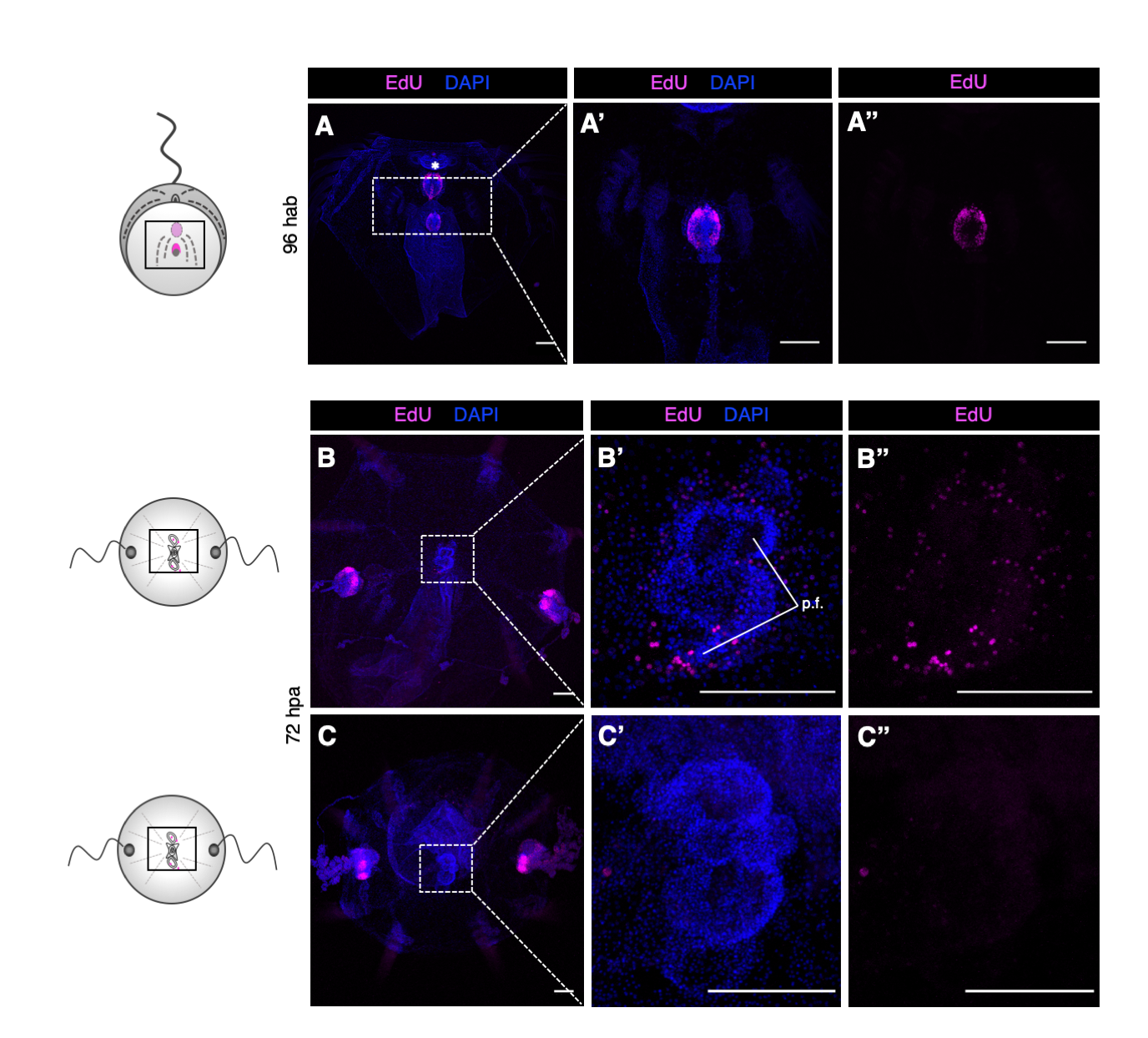

Supplement: Supplementary file 3 — Figure S6 and S7. EdU staining at 96 hab and 72 hpa. (A-A”) Confocal stack projections of a bisected cydippid through the oral-aboral axis at 96 hab oriented in a lateral view showing the cut site in the first plane. White asterisks point to tentacle bulbs of the uncut site. (B-C”) Confocal stack projections of cydippids in which the apical organ was amputated at 72 hpa. Examples of the two types of EdU pattern observed at this time-point are shown. Nuclei of S-phase cells are labeled with EdU (magenta) and all nuclei are counterstained with DAPI (blue). The pattern of EdU labeling corresponding to each time-point is shown in a cartoon on the left of the rows. Scale bars = 100 μm. Abbreviations: hours after bisection (hab), hours post amputation (hpa), polar field (pf). (TIFF 5018 kb) [file 12915_2019_695_MOESM3_ESM.tiff]

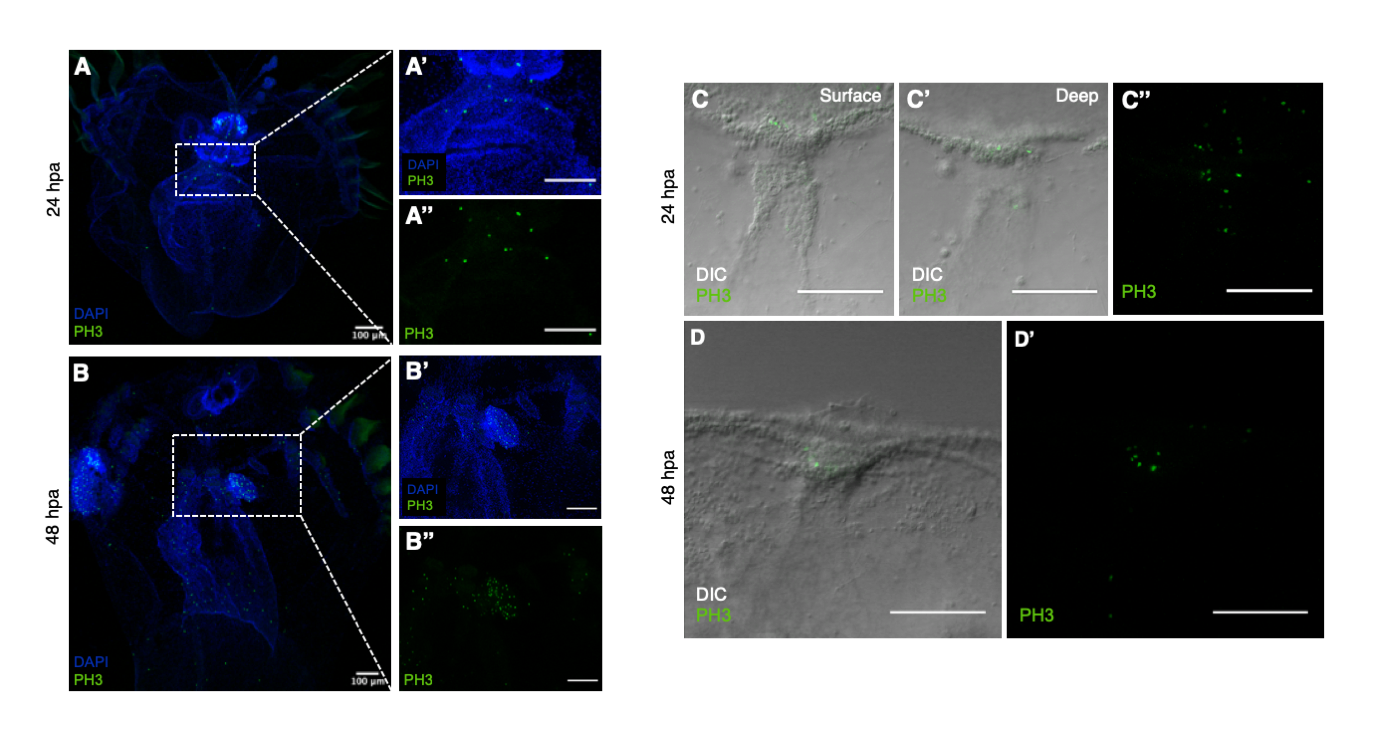

Supplement: Supplementary file 4 — Figure S6 and S7. PH3 immunostaining after oral-aboral bisection and apical organ amputation. (A-B”) Confocal stack projections of a bisected cydippid through the oral-aboral axis oriented in a lateral view showing the cut site in the first plane. The time following surgery is listed to the left of the rows. M-phase cells are stained with anti-PH3 (green) and all nuclei are counterstained with DAPI (blue). (C-D’) Confocal stack projections of regenerating apical organs in a lateral view. The time following surgery is listed to the left of the rows. M-phase cells are stained with anti-PH3 (green) combined with DIC image of the tissue. DIC images of surface and deep planes are shown for 24 hpa. Scale bars = 100 μm. Abbreviations: hours after bisection (hab), hours post amputation (hpa). (TIFF 2990 kb) [file 12915_2019_695_MOESM4_ESM.tiff]

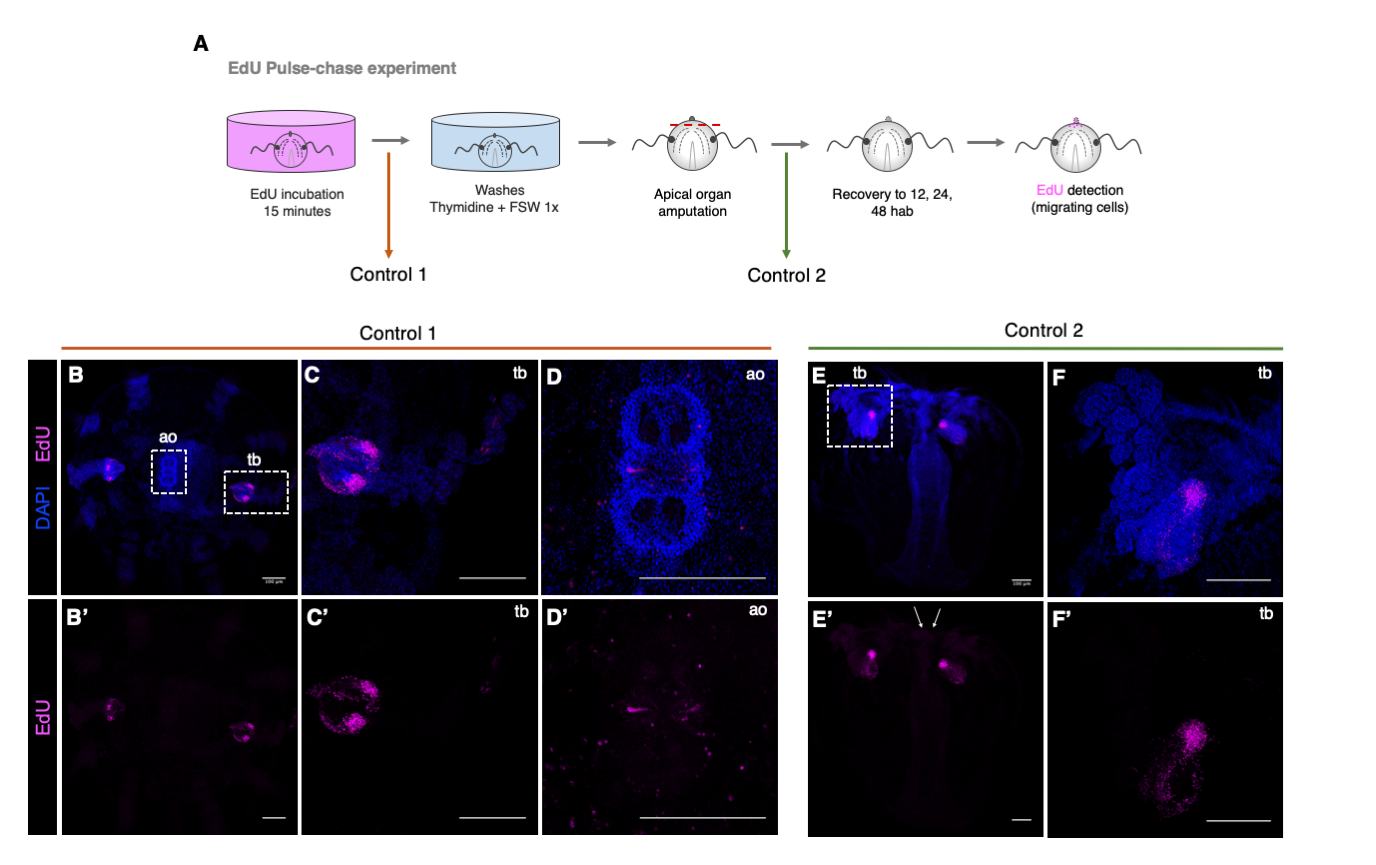

Supplement: Supplementary file 5 — Figure S8. Controls of the EdU pulse-chase experiments in regenerating cydippids. (A) Diagram of the experimental setup. (B-D’) Confocal stack projections of an uncut cydippid just after EdU incubation (control 1). (E-F’) Confocal stack projections of an amputated cydippid just after the cut (control 2). Nuclei of S-phase cells are labeled with EdU (magenta) and all nuclei are counterstained with DAPI (blue). White dotted rectangles in (B) and (E) show the area corresponding to higher magnifications on the right. White arrows in (E’) point to the wound site. Note that no EdU+ cells are detected at the wound site just after the cut while EdU staining is found in the normal areas of cell proliferation (tentacle bulbs). Scale bars = 100 μm. (TIFF 3490 kb) [file 12915_2019_695_MOESM5_ESM.tiff]

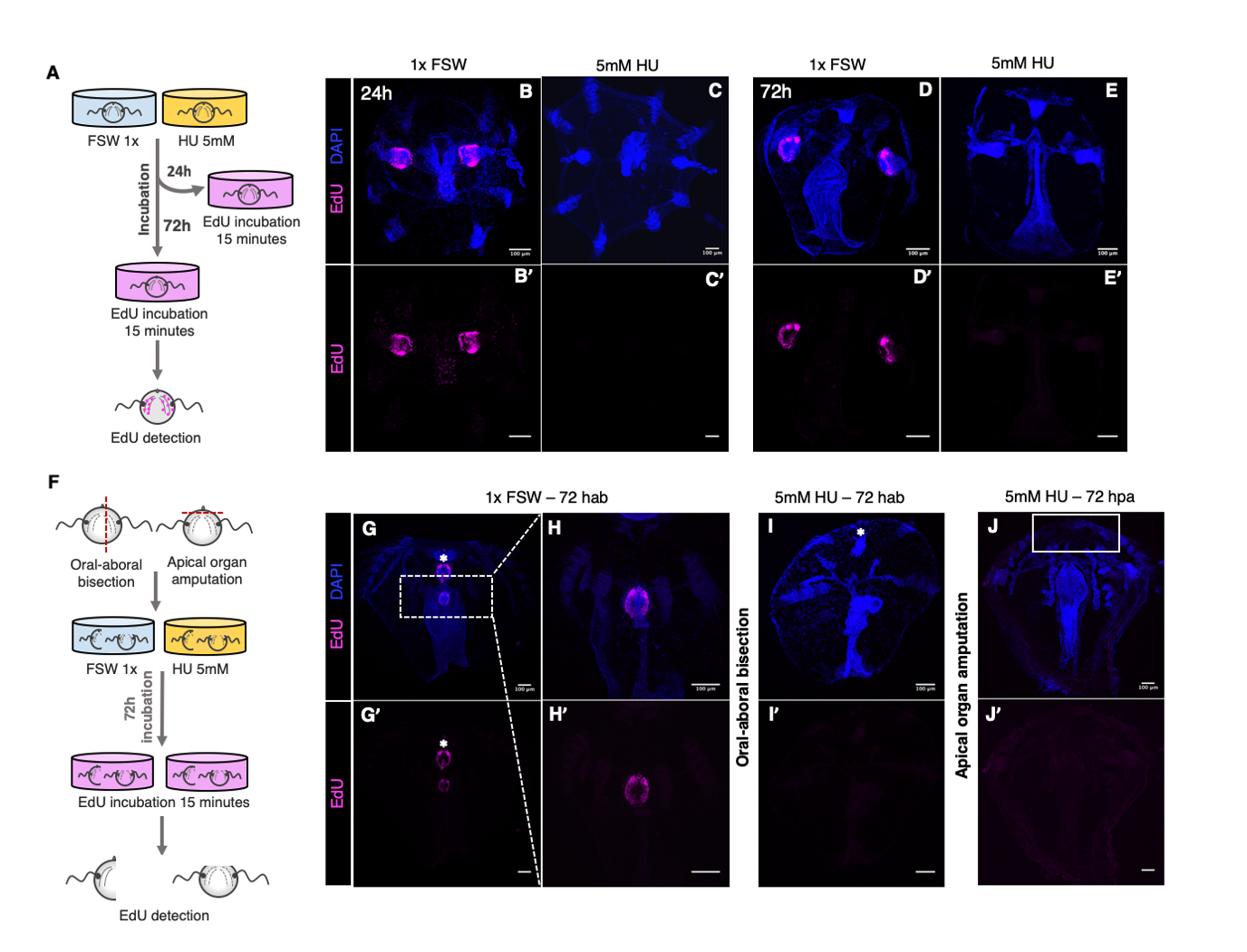

Supplement: Supplementary file 12 — Figure S11. 5 mM Hydroxyurea (HU) blocks cell proliferation in intact and bisected cydippids. (A) Schematic of cell proliferation inhibitor experiments with HU in intact cydippids. Confocal stack projections of intact cydippids oriented in an aboral view at 24 h (B-C’) and 72 h (D-E’) after HU incubation. The type of treatment is listed at the top of the columns, and the labeling corresponding to each panel is listed to the left of the rows. Nuclei of S-phase cells are labeled with EdU (magenta) and all nuclei are counterstained with DAPI (blue). Note that no EdU+ nuclei were detected in treated cydippids after HU incubation. (F) Schematic of cell proliferation inhibitor experiments with HU in dissected cydippids. (G-H′) Confocal stack projections of an untreated bisected cydippid oriented in a lateral view at 72 hab. (I-I′) Confocal stack projections of a bisected cydippids treated with HU at 72 hab. (J-J’) Confocal stack projections of cydippids amputated from the apical organ treated with HU at 72 hpa. The type of treatment and the time following bisection are listed at the top of the columns, and the labeling corresponding to each panel is listed to the left of the rows. The dotted line rectangle in (G) shows the area corresponding to higher magnifications on the right. The white asterisks in (G), (G’) and (I) point to the tentacle bulb of the uncut site. The white rectangle in (J) indicates the area of apical organ regeneration. Nuclei of S-phase cells are labeled with EdU (magenta) and all nuclei are counterstained with DAPI (blue). Note that no EdU+ nuclei were detected and none of the missing structures had regenerated in treated cydippids. Scale bars = 100 μm. Abbreviations: hours after bisection (hab), hours post amputation (hpa). (TIFF 4240 kb) [file 12915_2019_695_MOESM12_ESM.tiff]
